# Do Infants Really Learn Phonetic Categories?

**DOI:** 10.1162/opmi_a_00046

**Published:** 2021-11-01

**Authors:** Naomi H. Feldman, Sharon Goldwater, Emmanuel Dupoux, Thomas Schatz

**Affiliations:** Department of Linguistics and UMIACS, University of Maryland, College Park, MD, USA; School of Informatics, University of Edinburgh, United Kingdom; Cognitive Machine Learning (ENS - EHESS - PSL Research University - CNRS - INRIA), Paris, France; Facebook A.I. Research, Paris, France; Department of Linguistics and UMIACS, University of Maryland, College Park, MD, USA

**Keywords:** language acquisition, speech perception, computational modeling, representation learning

## Abstract

Early changes in infants’ ability to perceive native and nonnative speech sound contrasts are typically attributed to their developing knowledge of phonetic categories. We critically examine this hypothesis and argue that there is little direct evidence of category knowledge in infancy. We then propose an alternative account in which infants’ perception changes because they are learning a perceptual space that is appropriate to represent speech, without yet carving up that space into phonetic categories. If correct, this new account has substantial implications for understanding early language development.

## INTRODUCTION

**Figure 1.**
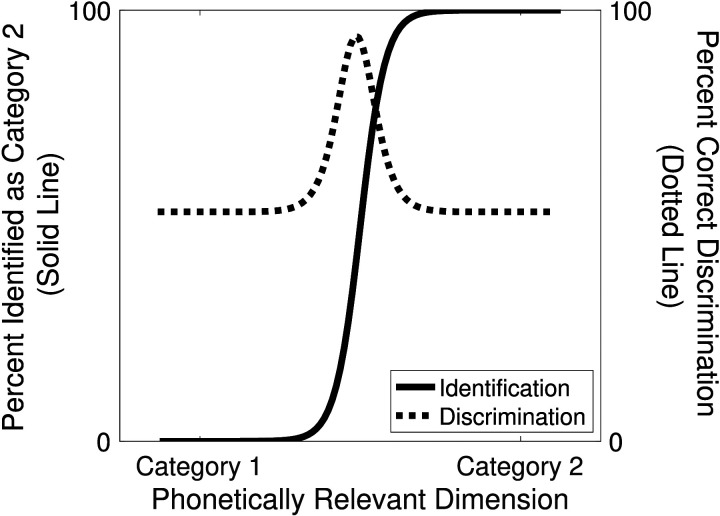
Hypothetical identification and discrimination functions in two-alternative forced choice tasks.

Infants’ perception of speech becomes specialized for the native language even before their first birthday. Discrimination of native contrasts improves, and discrimination of nonnative contrasts declines (Kuhl et al., 2006; Werker & Tees, 1984). These changes are often assumed to reflect the development of adultlike perceptual patterns, and more specifically of adultlike *phonetic category* representations: linguistically relevant categories that are phoneme-length and correspond roughly to the consonants and vowels of a language (Best, 1994; Kuhl et al., 1992; Werker et al., 2007; Zevin, 2012).^1^ These assumptions have been motivated by the close ties observed in adults between native language phonetic categories and language-specific patterns of discrimination along phonetically relevant dimensions, as shown schematically in Figure 1 (Liberman et al., 1957).

**Figure 2.**
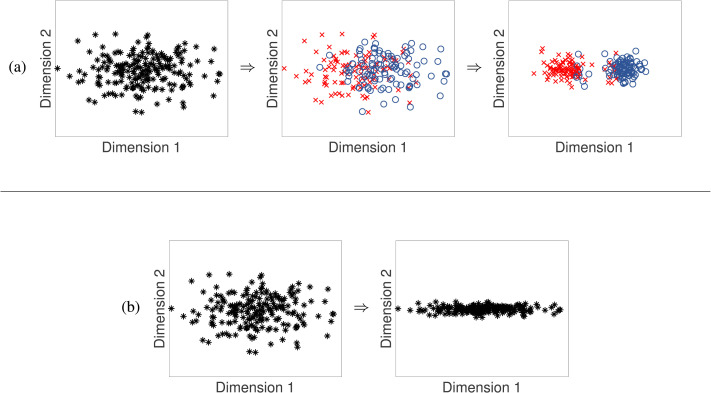
**Phonetic category learning vs. perceptual space learning.** (a) Under standard phonetic category learning theories, infants identify categories early. As a result, perception becomes warped along phonetically relevant dimensions (Dimension 1) and discrimination decreases along phonetically irrelevant dimensions (Dimension 2). (b) An alternative theory is that learners’ perceptual space undergoes substantial changes before phonetic categories are learned. In this simplistic example, perceptual learning collapses the dimension of lower variance, decreasing discrimination along Dimension 2. As described later, we believe perceptual space learning actually involves more complex transformations.

If early changes in discrimination result from early knowledge of phonetic categories—discrete units, with or without explicit labels, that roughly correspond to linguistically relevant sounds like [ɹ] (as in *rock*) and [l] (as in *lock*)—then infants must learn these categories by their first birthday. The categories would then drive changes to their perceptual space (Figure 2a). However, phonetic categories are difficult to learn from the speech infants hear (Antetomaso et al., 2017; Bion et al., 2013), raising doubts about the feasibility of early phonetic category learning. Early phonetic category learning has been questioned before (Jusczyk, 1992), yet only a few concrete alternative accounts of infants’ changes in discrimination have been proposed (Guenther & Gjaja, 1996; Herrmann et al., 1995; Matusevych et al., 2020; Schatz et al., 2021).

Here we critically examine the evidence for phonetic category learning in infancy and highlight recent developments in speech technology which, we argue, can inspire an alternative account of early perceptual learning where phonetic categories are not involved. Under this account, early changes in discrimination are caused by a learning process that—without recourse to phonetic categories—transforms the acoustic similarity space, changing the perceptual distances between sounds (Figure 2b). Phonetic categories are learned later, or more gradually, by carving up this learned space. We refer to the earlier phase of learning as *perceptual space learning*^2^ and discuss several algorithms that might be used to implement such learning, including learning without any discrete units, or with units that do not correspond meaningfully to phones. Changes in discrimination driven by knowledge of phonetic categories could in principle also be considered a type of perceptual space learning, but here we restrict the term to mean learning *without* phonetic categories. We do not argue conclusively *against* the early phonetic category learning hypothesis; instead, we argue that perceptual space learning, which has thus far received little attention in the language acquisition literature, should be seriously considered as a plausible alternative theory of what causes infants’ perceptual changes.

Attributing infants’ perceptual changes to perceptual space learning would have major implications for theories of language acquisition. Phonetic category learning has conventionally been thought to occur before (Werker et al., 2009) or alongside (Swingley, 2009) word learning, enabling word forms to be composed of sequences of phones from the earliest stages. This hypothesized trajectory makes phonetic category learning a difficult problem because it cannot draw on extensive knowledge of word meanings, which would provide information about which sounds in a language are meaningfully different (Trubetzkoy, 1939). However, if phonetic category learning occurs later in childhood, it could draw on a broad array of word meanings and minimal pairs, making it an easier problem (McMurray et al., 2018). Perceptual space learning would also have broad implications for other areas of language acquisition, such as understanding when and how infants notice that words are mispronounced (Curtin et al., 2009; Fennell & Werker, 2003; Rost & McMurray, 2009; Stager & Werker, 1997), studying whether infant-directed speech is optimized for phonetic learning (Cristia & Seidl, 2014; Eaves et al., 2016; Kuhl et al., 1997; McMurray et al., 2013), or understanding the challenges of adult second language learning (Flege & Hillenbrand, 1986; Francis & Nusbaum, 2002; Lipski et al., 2012; Underbakke et al., 1988; Ylinen et al., 2009). More generally, it would radically change our view of what children know at the beginning of their second year, a period when they rapidly acquire aspects of language related to grammar and meaning.

## CHILDREN’S PERCEPTUAL LEARNING

The primary evidence for phonetic category learning in infancy comes from experiments that measure infants’ discrimination of native and nonnative sound contrasts. The discrimination tasks do not inherently require category knowledge (Box 1), but they do reveal changes in discrimination that are suggestive of category learning (as articulated by Zevin, 2012). Discrimination of nonnative speech contrasts generally declines during the first year of life: by 10–12 months for consonants and by 6–8 months for vowels (Anderson et al., 2003; Best & McRoberts, 2003; Best et al., 1995; Bosch & Sebastián-Gallés, 2003; Burns et al., 2007; Kuhl et al., 1992; Segal et al., 2016; Tsuji & Cristia, 2014; Werker & Lalonde, 1988; Werker & Tees, 1984). During the same time period, discrimination of native contrasts generally improves (Burns et al., 2007; Kuhl et al., 2006; Narayan et al., 2010; Tsao et al., 2006). Although there are exceptions to this pattern (Best et al., 1988; L. Liu & Kager, 2014, 2016; Mattock & Burnham, 2006; Mazuka et al., 2014; Mugitani et al., 2009; Polka & Bohn, 1996; Polka et al., 2001; Sundara et al., 2006; Yeung et al., 2013), it is clear that infants’ perception becomes more native-like as they are exposed to their native language.

**Box 1.** **DO INFANT DISCRIMINATION TASKS REQUIRE CATEGORY KNOWLEDGE?** Most tests of infant speech perception have used one of two paradigms. In a *habituation* experiment, infants experience repeated trials in which they hear a habituation stimulus—exemplars from one phonetic category—while viewing a visual display. Once their looking time to habituation trials falls below a threshold, discrimination is measured as the extent to which they look longer at *change* trials (with exemplars from another category) than at *same* trials (with exemplars from the habituated category). Infants need to be able to discriminate a contrast in order to show different looking behavior toward *change* trials and *same* trials. However, infants can succeed at this task without knowing phonetic categories, as long as they perceive the stimuli on *change* trials to be acoustically anomalous, relative to the habituation trials. Similar considerations hold for the oddball paradigm used by Hochmann and Papeo (2014).

The other paradigm that is frequently used to measure infant speech perception is the *conditioned head turn* (CHT) procedure, in which infants face an experimenter who is playing with toys and hear a background stimulus from a loudspeaker on the side of the room. On *change* trials, the stimulus changes to an exemplar from the other phonetic category, and they can look toward the loudspeaker and see toys light up and start to move. On *same* trials, when the category does not change, looking toward the loudspeaker does not yield any visual reward. After an initial conditioning phase, discrimination is assessed by measuring head turns on *change* trials, relative to *same* trials. As in habituation experiments, infants need to be able to discriminate a contrast in order to show different looking behavior toward *change* trials and *same* trials. However, because this paradigm involves a decision of whether to perform a head turn, it resembles identification tasks in some ways. Particularly striking are studies showing that when trained on a phonetic contrast, infants can generalize to novel speakers during test in a CHT paradigm (Kuhl, 1979, 1983). This seems to suggest that infants already know that phonetic differences, but not speaker differences, signal a category distinction.

However, it is possible that the categorical patterns of generalization reflect learning that has occurred during the experiment. The visual reinforcements that infants see during a CHT experiment provide a reward signal that could engage reinforcement learning mechanisms, which appear to be particularly successful in driving auditory perceptual learning in adults (Lim et al., 2019; Lim & Holt, 2011; Tricomi et al., 2006). In line with this, Kuhl (1979) notes that the infants initially make head turns toward stimuli that vary from the background stimulus along irrelevant dimensions, such as speaker or pitch, but that this tendency lessens over the course of the experiment. She hypothesizes that learning has occurred during the experiment and suggests that

the infant demonstrates a proclivity to try to discover a criterial attribute which separates the two categories. The infant, in effect, displays a tendency to be a “natural sorter,” and is attracted to a dimension which makes a set of multidimensional auditory stimuli fit into easily recognized perceptual groupings. (p. 1674)

In other words, Kuhl hypothesizes that it is the functional equivalence of different exemplars with respect to the visual reinforcement in the CHT paradigm that supports learning of new cue weights. Given that this learning could occur within the experiment itself, the categorical head-turn behavior that infants exhibit within this paradigm does not necessarily support the strong hypothesis that they come into the lab with well-formed phonetic categories (see Apfelbaum & McMurray, 2011, for a similar argument). Whether, and at what age, children use the same strategy to learn phonetic categories in more naturalistic settings remains an open question.

A category-based account of these perceptual changes would entail that learners group stimuli into discrete units that correspond roughly to the phones of a language. As shown in Figure 2a, the categories would then drive changes in the perceptual space (Bonnasse-Gahot & Nadal, 2008; Kuhl, 1979). However, there are reasons to question whether categories are the driving force behind infants’ perceptual changes. Box 2 distinguishes three perceptual effects that are often associated with category knowledge. If all three are direct results of category knowledge, then they should develop in tandem, as categories are learned. Given the substantial evidence that discrimination of nonnative contrasts declines sharply relative to native contrasts during infants’ first year (Effect 3), one might also expect to find sharpening category boundaries (Effect 1) or sharpening discrimination peaks along phonetically relevant dimensions (Effect 2) in young infants. Yet there is little evidence that these effects develop during the same time period.

**Box 2.** **PERCEPTUAL EFFECTS ASSOCIATED WITH CATEGORIES** Three types of perceptual effects are typically assumed to arise from category knowledge. While there is substantial evidence that the first two are closely tied to knowledge of categories, or at least distinct clusters of sounds, we argue that the third effect is more general, and need not reflect such knowledge.

**Effect 1** is a sharp category boundary in identification tasks (Liberman et al., 1957; Figure 1). Performing an identification task requires category knowledge, given the use of category labels in the task. However, changes in steepness of the category boundary during learning could arise either from changes in category knowledge, or from children’s improving ability to perform an identification task. These two possibilities can be disambiguated through a phenomenon known as *cue weighting*, which refers to the relative steepness of the identification curve across different dimensions. Changes in cue weighting have been tied to category learning across many studies (Francis et al., 2000; Francis et al., 2008; Francis & Nusbaum, 2002; Holt & Lotto, 2006; Idemaru & Holt, 2011, 2014; Lehet & Holt, 2017; R. Liu & Holt, 2015; Yang & Sundara, 2019; Ylinen et al., 2009), and cue weights are also key to many models of categorization (Kruschke, 1992; Love et al., 2004; Nosofsky, 1986; Toscano & McMurray, 2010), suggesting that this effect is closely tied to category knowledge.

**Effect 2** is a discrimination peak near the category boundary (Liberman et al., 1957; Figure 1). While some models do attribute peaks in discrimination near the category boundary to category knowledge (Feldman et al., 2009; Kuhl, 1993; Lacerda, 1995), other models have suggested that this effect may only require distinct clusters in the distribution of sounds in the acoustic space (like the distributions in the third panel in Figure 2a) even if the clusters are not recognized as discrete units (Guenther & Gjaja, 1996; Herrmann et al., 1995; Shi et al., 2010). Moreover, categories with high variability (Figure 2a, second panel) may not yield a distinctive discrimination peak (Kronrod et al., 2016). Thus, we take the discrimination peak to index how tightly clustered the distribution of sounds is in listeners’ perceptual space. Whether well-separated clusters of sounds constitute perceptual categories is a matter of some debate; to avoid overloading terminology, we simply refer to these as clusters of sounds in a perceptual space.

**Figure 3.**
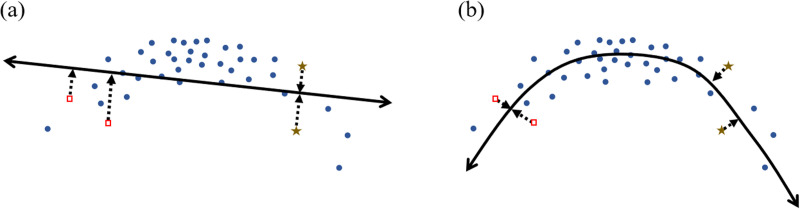
**Example illustrating how different perceptual space learning methods could lead to different perceived distances between the same original points.** Here, both methods map points from a two-dimensional space to a one-dimensional line. The mapping is shown explicitly for only four points; distances along the line correspond to perceptual distances in the learned space. (a) In the linear mapping, the brown stars are mapped to the same location, so the distinction between these points is lost, whereas the red squares remain distinct. (b) In the nonlinear mapping, the opposite holds.

**Figure 4.**
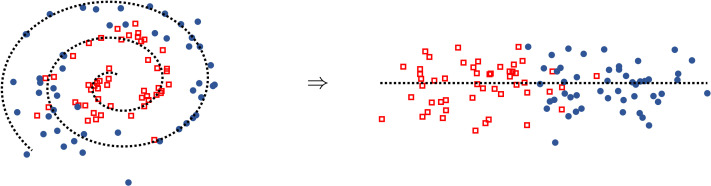
**Perceptual space learning can make category learning easier.** The marker shapes/colors represent ground truth category labels, which are unknown to the learner; the dotted line highlights the transformation. The decision boundary is simpler after transforming the space.

**Effect 3** is listeners’ differential ability to discriminate sounds along different dimensions. For example, English listeners discriminating instances of [ɹ] and [l] are more sensitive to differences in the third formant than to differences in the second formant, whereas Japanese listeners have roughly equal sensitivity to both dimensions (Iverson et al., 2003). Listeners can retain sensitivity to cues even when they stop using those cues to categorize sounds (Lehet & Holt, 2020), so changes in sensitivity in discrimination tasks are not necessarily the same thing as changes in cue weighting. In theory, it is possible to lose or gain the ability to discriminate along certain dimensions even without representing well-separated clusters of sounds in a perceptual space (Figure 2b; Figure 3; Figure 4); that is the possibility we explore in this article.

The scope of this last effect merits consideration, because although discrimination is typically assumed to be better along phonetically relevant dimensions than along phonetically irrelevant dimensions (cf. Goldstone, 1994), there are exceptions to this generalization (Best et al., 1988). Moreover, even if there were no exceptions, predicting exactly which contrasts are difficult to discriminate requires knowing the dimensions of listeners’ perceptual space. The second formant in tokens of [l] or [r] may be a different perceptual dimension than the second formant in vowels, for instance. For the purposes of this article, we take the primary signature of Effect 2 to be a peak in discrimination near a category boundary. Absent evidence of the development of such a peak, we tentatively assume that any changes in discrimination could instead be instances of Effect 3.

Identification tasks are challenging to carry out with infants, but the few studies that have directly measured English-learning infants’ categorization have found extremely shallow identification boundaries (Burnham, 1986; Burnham et al., 1991). Boundaries become steeper—as measured through aggregated data and individual participants’ identification functions—between 3 and 7 years, with differences even between 6- or 7-year-olds and adults in some cases (Burnham, 1986; Burnham et al., 1991; Chen et al., 2017; Hazan & Barrett, 2000; Krause, 1982; Kuijpers, 1996; McMurray et al., 2018; Ohde & Haley, 1997; Simon & Fourcin, 1978; Zlatin & Koenigsknecht, 1975). These changes could be partly due to children’s improving ability to perform identification tasks, but task difficulty is not the only factor. Across much of the range between 3-year-olds and adults, the increase in category boundary steepness depends on the category being tested (Slawinski & Fitzgerald, 1998) and on the specific phonetic dimensions along which those categories are tested (Greenlee, 1980; Hazan & Barrett, 2000; Nittrouer, 1992; Nittrouer & Miller, 1997; Nittrouer & Studdert-Kennedy, 1987; Ohde et al., 1995; Ohde & Haley, 1997), indicating that children are reweighting different dimensions as cues to category membership. These differential changes in category boundary steepness strongly suggest that at least some category learning occurs later in childhood.

Discrimination peaks along phonetically relevant dimensions sharpen in tandem with the changes in category boundary steepness later in childhood (Chen et al., 2017; Medina et al., 2010), whereas in infants, evidence for the development of discrimination peaks is mixed. Newborn and 6-month-old English and Swedish learners show cross-linguistic differences in vowel perception for [i] and [y] (Kuhl et al., 1992; Moon et al., 2013), and English-learning 6-month-olds’ discrimination is worse near a prototypical [i] than near a nonprototypical [i], similar to adults (Grieser & Kuhl, 1989; Kuhl, 1991). These studies are suggestive, but do not provide direct evidence that between-category discrimination peaks are developing in infancy. In consonants, there are cross-linguistic differences in infants’ voice onset time (VOT) discrimination (Eilers et al., 1979; Streeter, 1976), with a clear peak in discrimination near the phonetic category boundary in English-learning 1- and 4-month-old infants (Eimas et al., 1971). However, a meta-analysis of infant studies with English learners did not find evidence that the VOT discrimination peak sharpens over the first year of life (Galle & McMurray, 2014). Moreover, the discrimination peak is also present in nonhuman animals (Kuhl, 1981; Kuhl & Miller, 1975; Kuhl & Padden, 1982), suggesting that it arises from an auditory discontinuity. Whether auditory discontinuities constitute knowledge of categories, and how they relate to subsequent perceptual learning, is less clear (Chládková & Paillereau, 2020). One study did find that French-learning infants’ VOT discrimination changes between 4 and 8 months in the direction that would be expected if they were learning phonetic categories (Hoonhorst et al., 2009), providing some evidence of a developing discrimination peak. Overall, however, there is little convincing evidence that peaks in discrimination along phonetically relevant dimensions sharpen substantially during infants’ first year.

The literature thus suggests that different perceptual changes occur at different ages. Infants’ discrimination changes substantially during the first year (Effect 3), but changes that are diagnostic of category learning (Effect 1) and of increasing perceptual separation between clusters of sounds (Effect 2) are most clearly documented later in childhood. Existing accounts nevertheless attribute both infant and childhood perceptual changes to category learning (Burnham, 1986; Zevin, 2012). We question this interpretation for two reasons. First, as we argue in the next section, general changes in discrimination are compatible with various perceptual space learning algorithms that do not require phonetic categories at all. Second, for phonetic categories to be the cause of those drastic early perceptual changes, one must either posit well-developed categories (in which case the missing evidence of Effect 2 is puzzling), or suppose that noisy, poorly developed categories can drive a drastic reshaping of the perceptual space to yield Effect 3, even though those same category representations are too noisy to yield discrimination peaks along phonetically relevant dimensions (Effect 2).

For these reasons, we believe it is time for the field to consider the possibility that infants’ perceptual changes primarily reflect a perceptual space learning process. Early perceptual development would look more like Figure 2b, or a more sophisticated variant (discussed following). Learning phonetic categories to carve up this perceptual space could then extend well into childhood and even adolescence. Although there is, as yet, little empirical evidence to distinguish this hypothesis from the early phonetic category learning hypothesis, the latter makes stronger assumptions about the nature of early representations that have yet to be clearly validated.

## COMPUTATIONAL APPROACHES TO PERCEPTUAL SPACE LEARNING

Although cognitive scientists have proposed a handful of perceptual space learning models for speech (Gauthier et al., 2007; Guenther & Gjaja, 1996; Herrmann et al., 1995; Nixon & Tomaschek, 2021; Westermann & Reck Miranda, 2004), perceptual space learning is more actively studied in the machine learning community, where it is well-known that modified representations of input features can be learned without access to, and without necessarily resulting in, categorical knowledge. This type of learning has been used in many domains, including vision and speech (Chung et al., 2019; Erhan et al., 2010; Kamper et al., 2015; Ranzato et al., 2007; Schneider et al., 2019; van den Oord et al., 2018; Yu et al., 2010), and there is even a series of recent speech technology challenge tasks devoted to the topic (Dunbar et al., 2017, 2019; Versteegh et al., 2015).

Perceptual space learning is popular in machine learning because it can improve a system’s ability to learn from the signal: for example for speech, spectral information, or even waveforms. In contrast, cognitive models often use more abstract features (such as formants) as input. However, starting from abstract features skips over a critical part of the learning process, wherein infants must learn which of the many dimensions of raw speech are relevant to processing their native language. We argue that this aspect of learning, which most cognitive models do not consider at all, could explain many of the perceptual changes seen in young infants.

To illustrate, consider a well-known method for perceptual space learning: principal component analysis (PCA). PCA reduces the dimensionality of data in order to learn a more compact representation that still preserves the most important information. For example, in the speech domain each input data point might represent a short (10 ms) slice of speech using a vector where each dimension represents the value of some acoustic measure such as spectral energy. Some of these dimensions may vary independently, while others may be highly correlated or simply record random noise—thus, most of the information can be represented using a smaller number of dimensions. PCA identifies the orthogonal dimensions of greatest variation in the original data, rotates these to align with the axes of the vector space, and discards dimensions with low variation. That is, it learns a representation that is optimized to capture the greatest amount of variance in the data.

The transformation learned by PCA is *linear*, since it simply rotates the axes of the space before collapsing some dimensions. However, many perceptual space learning methods are more powerful, in that they learn a *nonlinear* transformation, warping the original space in potentially arbitrary ways (Figure 3).^3^ The result is that points that were close together in the input space may end up far apart in the learned space or vice versa. Therefore, if discrimination depends on distance in some perceptual space (Shepard, 1987), perceptual space learning could lead to changes in discrimination.

Although perceptual space learning is not directly optimized for categorization, it could nevertheless help with later category learning by factoring out irrelevant features or warping the space in a way that makes the category structure more obvious (Figure 4). This effect has been demonstrated both in cognitive models of auditory learning (Gauthier et al., 2007; Roark et al., 2020) and in machine learning models, where “pretraining” a system’s perceptual space on a generic unsupervised task (such as predicting the next input in a sequence) can improve performance on a variety of downstream tasks (such as question answering or phone classification) (Chung et al., 2019; Devlin et al., 2019; Erhan et al., 2010; Peters et al., 2018; Schneider et al., 2019). While it is theoretically possible that systems pretrained on speech could be implicitly learning phonetic categories, evidence from models that do learn quantized representations (latent categories) suggests otherwise: the learned units are typically far more granular than phonetic categories, and often cannot even be well-characterized as sub-phones or subsets of phonetic categories (Baevski et al., 2020; Baevski, Schneider, & Auli, 2019; Chorowski et al., 2019; Hsu et al., 2021; Schatz et al., 2021).

These recent successes in machine learning have led to a proliferation of new work on perceptual space learning algorithms. Thus, cognitive scientists should be considering not just whether perceptual space learning could explain infants’ early perceptual development, but more specifically which algorithms might provide good models for infant learning. These algorithms differ in the source of the learning signal and the cognitive plausibility and domain-specificity of the mechanism. For example, self-organizing maps (Kohonen, 1989, 2001) are an early method for nonlinear dimensionality reduction, based on competitive learning. More popular in the speech community are autoencoder neural networks (Chorowski et al., 2019; van Niekerk et al., 2020), which can be viewed as a domain-general learning mechanism inspired by memory encoding: they learn to encode each input into an internal representation that allows the original input to be reconstructed as closely as possible. Other recent algorithms aim to predict missing or upcoming stretches of speech, with the learning signal coming from prediction errors—another cognitively plausible domain-general mechanism (Baevski, Auli, & Mohamed, 2019; Baevski et al., 2020; Baevski, Schneider, & Auli, 2019; Chung et al., 2019; Hsu et al., 2021).

There have also been recent proposals for more domain-specific perceptual space learning methods that rely on a noisy top-down signal provided by knowledge of some word-like units (Kamper et al., 2015; Renshaw et al., 2015; Riad et al., 2018; Thiollière et al., 2015). These units can be found by searching for stretches of speech that form similar pairs or clusters, without any knowledge of phones (Jansen & Van Durme, 2011; McInnes & Goldwater, 2011; Park & Glass, 2008; Räsänen & Blandon, 2020). Assuming that the clusters represent different instances of the same word, the learner can then adjust its current representation of the low-level speech features to make these instances even closer together in perceptual space. Preliminary evidence suggests that models using this mechanism can learn representations that demonstrate some of the effects seen in infants (Matusevych et al., 2020). At a high level, this is essentially the mechanism proposed by Jusczyk (1992), and—unlike the other methods described above—it does use a form of categorical knowledge (word categories) to guide learning. Whereas we argue in the next section that phonetic categories are difficult to learn due to high acoustic overlap, word-like units are likely to have fewer near acoustic neighbors than phones (Swingley, 2009), which could make them easier for infants to discover in naturalistic speech (cf. Jusczyk & Aslin, 1995; Jusczyk et al., 1999).

## REVISITING PHONETIC CATEGORY LEARNING

Learners eventually develop sharp identification boundaries and discrimination peaks, providing evidence of well-separated categories (Box 2). Under a phonetic category learning account of infants’ perceptual changes, much of the category learning process happens in infancy. Under a perceptual space learning account, category learning might occur later or more gradually, and even if it begins in infancy, it is not the primary driver of infants’ perceptual changes. Either way, there must be a mechanism for learning phonetic categories.

*Distributional learning* (Maye et al., 2002) has emerged as a leading hypothesis for a mechanism that could operate in infancy. Infants discriminate stimuli better after hearing a bimodal distribution—with two distinct clusters of sounds—along the relevant phonetic dimension than after hearing a unimodal distribution (Cristia, 2011; Maye et al., 2002; Maye et al., 2008; Wanrooij et al., 2014; Yoshida et al., 2010; see Cristia, 2018, for a meta-analysis). This ability to track acoustic distributions of sounds could support category learning if phonetic categories corresponded to well-separated clusters of sounds.

However, while some contrasts in laboratory speech are well-separated acoustically (Lisker & Abramson, 1964), categories overlap substantially in naturalistic speech, as in the second panel of Figure 2a (Antetomaso et al., 2017; Bard & Anderson, 1982; Bion et al., 2013; Hitczenko et al., 2020; Pollack & Pickett, 1963; Swingley, 2019).^4^ Most models that have tested the feasibility of distributional learning for identifying phonetic categories have simplified the learning problem, for example, by using artificial data with low variability (McMurray et al., 2009; Pajak et al., 2013; Vallabha et al., 2007), focusing only on subsets of the categories infants would need to acquire (Adriaans & Swingley, 2017; de Boer & Kuhl, 2003; Gauthier et al., 2007), or limiting the training data to a single speaker (Miyazawa et al., 2010; Miyazawa et al., 2011). Similar models that were tested on more realistic datasets showed much worse performance at learning phonetic categories (Adriaans & Swingley, 2012; Jones et al., 2012; Schatz et al., 2021). Therefore, the distributional sensitivity that infants exhibit in simplified laboratory settings may not be sufficient to learn phonetic categories in naturalistic settings. This may still be true even after perceptual space learning (as in the second panels of Figure 2b and Figure 4).

Aside from distributional information, phonetic category learners can draw on additional sources of information, such as word forms or meanings (Swingley, 2009). Infants recognize word forms in fluent speech (Bortfeld et al., 2005; Jusczyk & Aslin, 1995; Jusczyk et al., 1999) and know some word meanings (Bergelson & Swingley, 2012); both can affect infants’ discrimination in laboratory settings (Feldman, Myers, et al., 2013; Yeung & Werker, 2009). However, unsupervised phonetic category learning models that use contextual information have again done better when trained in idealized settings than in more naturalistic settings (Antetomaso et al., 2017; Feldman et al., 2013; Frank et al., 2014; C.-Y. Lee et al., 2015).

These differences between naturalistic and idealized settings make category-based accounts of infants’ perceptual changes less parsimonious than previously believed. When categories are heavily overlapping along some dimensions, as in the second panel of Figure 2a, separating them—even imperfectly, as in the third panel of Figure 2a—requires finding better dimensions for representing the sounds in the underlying perceptual space. Such a transformation is similar to perceptual space learning, but is driven by category knowledge. Thus, both the category-based account and the perceptual space learning account require the same two learning processes. What is at stake is the interdependence and relative timing of those processes. If phonetic category learning is as difficult as the above evidence suggests, it might be more feasible for older children, who can draw on more knowledge of higher level linguistic structure (McMurray et al., 2018) and benefit from using a learned perceptual space with fewer irrelevant dimensions.

## EMPIRICAL EVIDENCE FOR PERCEPTUAL SPACE LEARNING

There is not yet any direct evidence for a perceptual space learning process in infants. However, evidence from adults lends plausibility to such an account. After hearing nonspeech stimuli in which two auditory dimensions are perfectly correlated, listeners can discriminate between stimuli that follow the same correlation as in training, but not those that violate the correlation (Stilp et al., 2010; Stilp & Kluender, 2012), suggesting that correlations among dimensions can drive auditory perceptual space learning. The integration of perceptual dimensions for perceiving speech is not always determined by experience (Kingston et al., 2008; S. Lee & Katz, 2016), but several studies have suggested that an experience-based perceptual space learning process could play a role (Holt et al., 2001; Nearey, 1997; Schertz et al., 2020) and could interact in nontrivial ways with subsequent learning of cue weights (Roark et al., 2020; Roark & Holt, 2019; Scharinger et al., 2013).

Adults are additionally sensitive to temporal structure *within* perceptual dimensions. Their attention to dimensions in visual perception, such as color or shape, is affected by the temporal statistics within each dimension (Zhao et al., 2013)—that is, conditional probabilities, which infants are sensitive to in auditory perception (Saffran et al., 1996). This attentional benefit may well have an analogue in the auditory domain, given that auditory exposure to temporal regularities elicits increased MEG amplitude in auditory cortex relative to random sequences (Barascud et al., 2016). Although there is not yet evidence linking this attentional benefit of temporal structure to infants’ early perceptual changes, such a strategy could potentially be effective at identifying informative perceptual dimensions, because language has considerable internal structure.

## THE WAY FORWARD

To begin testing which type of theory best accounts for early perceptual development in speech, it is important to take seriously the complexity of speech produced in naturalistic environments. Naturalistic speech varies along many more acoustic dimensions than are typically manipulated in stimuli for speech perception experiments, or represented in phonetic learning models, and several studies have already shown that considering the variability of naturalistic speech can change our understanding of perceptual development (Antetomaso et al., 2017; Bion et al., 2013; Hitczenko et al., 2020). Methods for working with speech in naturalistic settings have been developed in the context of engineering applications, and naturalistic speech corpora now exist in numerous languages. By adapting these tools (e.g., Räsänen, 2011; Räsänen & Rasilo, 2015; Schatz, 2016; Schatz et al., 2013, 2021; Schatz et al., 2018; Schatz & Feldman, 2018), cognitive scientists can begin investigating the role of perceptual space learning in explaining how infants’ perception of speech becomes specialized for their native language.^5^

Thus far, we know of only a handful of models that have been evaluated against infant behavioral data after training on natural continuous speech. Schatz et al. (2021) trained a bottom-up distributional learner—specifically, a Dirichlet process Gaussian mixture model—on low-level spectral representations of speech from Japanese or English. The model reproduced infants’ discrimination of [ɹ] and [l], but the units it learned did not resemble phonetic categories. Matusevych et al. (2020) found that a recurrent neural network that optimized its hidden representations to represent correspondences between tokens of the same word achieved performance comparable to the model from Schatz et al. (2021). The success of these models suggests that alternatives to the phonetic category learning hypothesis, including perceptual space learning models that have no sub-word categories at all, are well worth exploring. In contrast, we are not aware of a phonetic category–based model that has been trained on continuous, unsegmented speech and used to predict cross-linguistic patterns of infants’ discrimination (see Schatz et al., 2021, supplementary discussion 1, for further discussion of this gap in the literature).

Parallels between the phonetic learning and machine learning literatures provide other reasons to be optimistic about perceptual space learning theories. Perceptual space learning algorithms that rely on word-like units (Kamper et al., 2015; Renshaw et al., 2015; Riad et al., 2018; Thiollière et al., 2015) are reminiscent of proposals that the words infants segment from fluent speech can constrain phonetic category learning (Feldman, Griffiths, et al., 2013; Swingley, 2009). The distributional learning strategy that Schatz et al. (2021) used is similar to that proposed by Maye et al. (2002) to learn phonetic categories. Both of these strategies have struggled to scale to more realistic data under a phonetic category learning account (Antetomaso et al., 2017; Bion et al., 2013; Taniguchi et al., 2016), but perform well once the constraint that phonetic categories need to be learned is dropped.

Jusczyk (1992) proposed over 25 years ago that phonetic learning might not rely on phonetic categories, but this idea has largely been disregarded in the literature on phonetic learning. Here we have argued that this idea is consistent with a large body of empirical literature on infant phonetic learning and have connected the proposal to recent trends in speech technology that provide paths toward a formal theory. The time course of phonetic category learning has major implications for our understanding of language acquisition as a whole, and as such we hope this article will inspire serious consideration of the perceptual space learning hypothesis and encourage the kind of rigorous empirical and computational tests that can ultimately distinguish it from the currently popular alternative.

## ACKNOWLEDGMENTS

We thank Adam Albright, Richard Aslin, Yevgen Matusevych, Bob McMurray, and two anonymous reviewers for insightful comments.

## FUNDING INFORMATION

NHF, National Science Foundation (https://dx.doi.org/10.13039/100000001), Award ID: BCS-1734245. SG, Economic and Social Research Council (https://dx.doi.org/10.13039/501100000269), Award ID: ES/R006660/1. SG, James S. McDonnell Foundation (https://dx.doi.org/10.13039/100000913), Award ID: Scholar Award 220020374. ED, Agence Nationale pour la Recherche, Award ID: ANR-17-EURE-0017 Frontcog. ED, Agence Nationale pour la Recherche, Award ID: ANR-10-IDEX-0001-02 PSL*. ED, Agence Nationale pour la Recherche, Award ID: ANR-19-P3IA-0001 PRAIRIE 31A Institute. ED, Facebook AI Research, Award ID: Research Grant.

## AUTHOR CONTRIBUTIONS

NHF: Conceptualization: Lead; Funding acquisition: Lead; Investigation: Lead; Writing – original draft: Lead; Writing – review & editing: Lead. SG: Conceptualization: Lead; Funding acquisition: Lead; Investigation: Lead; Writing – original draft: Lead; Writing – review & editing: Lead. ED: Conceptualization: Supporting; Funding acquisition: Supporting; Writing – review & editing: Supporting. TS: Conceptualization: Supporting; Writing – review & editing: Supporting.

## Notes

^1^ Contextual variants of a phoneme are generally treated as different categories, with phonetic categories corresponding roughly to allophones (Dillon et al., 2013; Werker & Curtin, 2005; but see Pegg & Werker, 1997).^2^ In machine learning the usual term is *unsupervised representation learning*, but we want to avoid confusion caused by the broader meaning of *representation* in cognitive science.^3^ Although both of the illustrated methods reduce dimensionality, perceptual space learning can also maintain or even increase dimensions; the key property is that it changes the shape of the input space.^4^ Although the degree of overlap depends on the specific dimensions measured, we know of no language-universal set of dimensions that reliably yields well-separated phonetic categories (see also Chládková & Paillereau, 2020).^5^ A complete model would also need to incorporate social factors (Conboy et al., 2015; Kuhl et al., 2003; Lytle et al., 2018; Tripp et al., 2021).
